# Ultra-Stable Sodium-Ion Battery Enabled by All-Solid-State Ferroelectric-Engineered Composite Electrolytes

**DOI:** 10.1007/s40820-024-01474-6

**Published:** 2024-07-25

**Authors:** Yumei Wang, Zhongting Wang, Xiaoyu Xu, Sam Jin An Oh, Jianguo Sun, Feng Zheng, Xiao Lu, Chaohe Xu, Binggong Yan, Guangsheng Huang, Li Lu

**Affiliations:** 1 College of Aerospace Engineering, Chongqing University, Chongqing, 400044 People’s Republic of China; 2 National University of Singapore (Chongqing) Research Institute, Chongqing, 401123 People’s Republic of China; 3 College of Materials Science and Engineering, Chongqing University, Chongqing, 400044 People’s Republic of China; 4 Department of Mechanical Engineering, National University of Singapore, 9 Engineering Drive 1, Singapore, 117575 Singapore; 5 Fujian Key Laboratory of Special Energy Manufacturing, Xiamen Key Laboratory of Digital Vision Measurement, Huaqiao University, Xiamen, 361021 People’s Republic of China

**Keywords:** Sodium-ion battery, NVP anode, All-solid-state, Cyclic stability, Ferroelectric

## Abstract

Symmetric Na-ion cells using the NASICON-structured electrodes could simplify the manufacturing process, reduce the cost, facilitate the recycling post-process, and thus attractive in the field of large-scale stationary energy storage. However, the long-term cycling performance of such batteries is usually poor. This investigation reveals the unavoidable side reactions between the NASICON-type Na_3_V_2_(PO_4_)_3_ (NVP) anode and the commercial liquid electrolyte, leading to serious capacity fading in the symmetric NVP//NVP cells. To resolve this issue, an all-solid-state composite electrolyte is used to replace the liquid electrolyte so that to overcome the side reaction and achieve high anode/electrolyte interfacial stability. The ferroelectric engineering could further improve the interfacial ion conduction, effectively reducing the electrode/electrolyte interfacial resistances. The NVP//NVP cell using the ferroelectric-engineered composite electrolyte can achieve a capacity retention of 86.4% after 650 cycles. Furthermore, the electrolyte can also be used to match the Prussian-blue cathode Na_*x*_Fe_*y*_Fe(CN)_6−*z*_·nH_2_O (NFFCN). Outstanding long-term cycling stability has been obtained in the all-solid-state NVP//NFFCN cell over 9000 cycles at a current density of 500 mA g^−1^, with a fading rate as low as 0.005% per cycle.

**Supplementary Information:**

The online version contains supplementary material available at 10.1007/s40820-024-01474-6.

## Highlights

- The capacity fading mechanism of the conventional Na_3_V_2_(PO_4_)_3_//Na_3_V_2_(PO_4_)_3_ (NVP//NVP) cell has been investigated.
- All-solid-state ferroelectric-engineered composite electrolyte could improve the electrolyte–electrode interfacial stability as well as the interfacial ion conduction of the Na-ion battery using the NVP anode.
- Outstanding cyclic stability has been achieved in the all-solid-state Na-ion battery using the NVP anode, with a capacity fading rate as low as 0.005% per cycle.

**Supplementary Information:**

The online version contains supplementary material available at 10.1007/s40820-024-01474-6.

## Graphical abstract

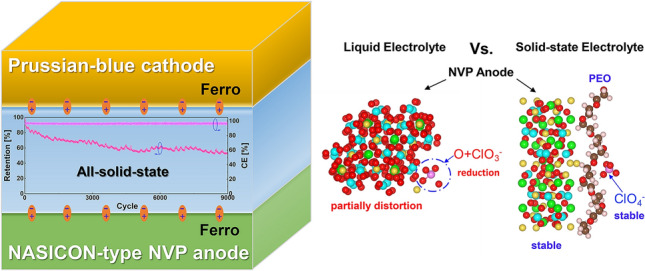

## Introduction

**Fig. 1:**
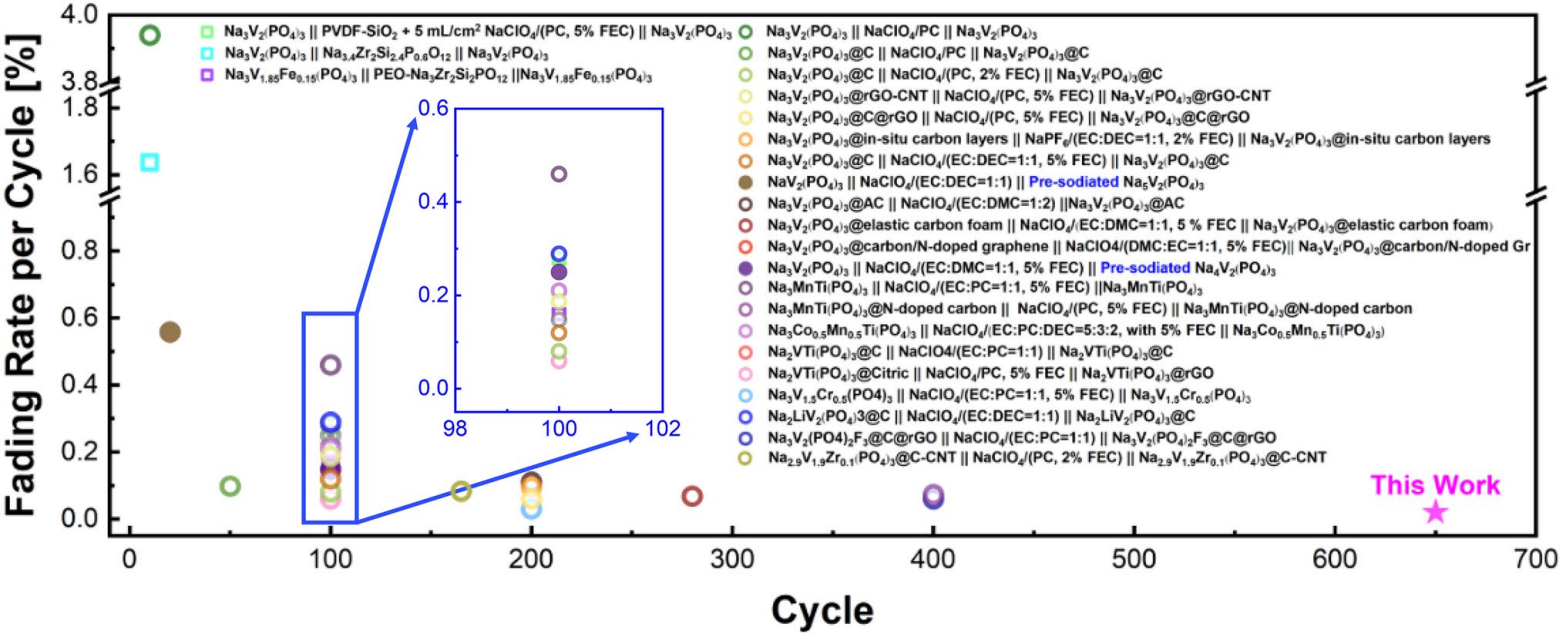
Cyclic stability of the symmetric sodium-ion batteries using NASICON-structured electrodes

Although sodium-ion battery has relatively low specific energy density compared to that of the lithium-ion battery, the sodium-ion battery possesses long-term stable cyclability and low processing cost due to the crystalline structure of the electrode materials and the high abundance of the sodium resources [1–3]. As one of the most acceptable electrodes, the NASICON-structured Na_3_TM_2_(PO_4_)_3_ (TM, transition metals such as V and Ti), has been extensively investigated. With two redox couples of the TM, it can therefore be used as a cathode as well as an anode forming symmetric cells. The design of symmetric cells could further simplify the manufacturing process, reduce the cost and facilitate the recycling post-process. Furthermore, the symmetric cells using the same electrode material could reduce the bulk expansion of electrodes during cycling because one electrode shrinks while the other electrode would expand. [4–16] Therefore, the symmetric cells are highly attractive in the field of large-scale stationary energy storage. As a typical NASICON-structured electrode, Na_3_V_2_(PO_4_)_3_ shows excellent stability when tested using its high redox potential (V^4+/3+^); however, low cyclic stability has been widely reported when V^4+/3+^ and V^2+/3+^ are both used in the symmetric cells. As summarized in Fig. 1, the discharge capacity fades quickly in those symmetric cells using the liquid electrolytes (represented by the circle symbol), within which the cost-effective NaClO_4_ is selected as the sodium salt. The capacity fading rate of these cells is as high as ~ 3.94% per cycle [13–34]. It is believed that the poor kinetics of the anode and the formation of undesired solid electrolyte interphase (SEI) on the anode lead to the quick capacity fading [13, 28]. Therefore, improvement and creation of stable SEI on the anode are important and necessary. However, it is noted that many trails through modification of the current liquid organic electrolytes or the formation of designed SEI are unable to well address the issue of capacity fading. The fading rate could only be improved to 0.07% per cycle (as shown in Fig. 1). On the other hand, the liquid electrolyte always raises severe safety concerns, due to its leakage, volatilization, and high flammability. Hence, it becomes crucial to develop new electrolytes that can not only enhance the cyclability of sodium-ion batteries by stabilizing SEI but also improve safety of the battery cells.

One of the solutions to address is to develop solid-state electrolytes as so to solid-state batteries. However, based on our best knowledge, the current state-of-the-art solid-state batteries could only deliver about 45%–90% theoretical discharge capacity with a fading rate of ~ 0.16% per cycle (the square symbol in Fig. 1) [35–37], and it is impossible to use high mass-loading electrodes, limiting the full utilization of the capacity of electrode materials, because sodium ions are unable to readily migrate within the electrode without enough ionic conduction medium. On the other hand, poor kinetics of Na^+^ within solid electrolytes often degrades the cyclability of the batteries made of solid electrolytes as the bridge for Na^+^ transportation [11]. Furthermore, the contact between rigid solid electrolyte and electrode is usually poor, limiting the battery performance. In the symmetric sodium-ion battery using the ceramic electrolyte, the capacity fading rate is above 1.6% per cycle, even though the ceramic electrolyte already possesses a high ionic conductivity (~ 10^–3^ S cm^−1^ at room temperature) [35]. During charge/discharge, high interfacial stresses between the rigid ceramic electrolyte and electrodes are generated due to the volume change of electrodes which may lead to delamination if the interfacial stresses exceed the bounding strength, and finally to the capacity fade. To minimize the interfacing stresses and hence suppress delamination, flexible polymer-based electrolyte can be an alternative. The well-designed polymer-based composite electrolytes possess reasonably good conductivity of about 10^–4^ S cm^−1^ at room temperature and most importantly have good flexibility to maintain the intimate contacts with electrodes [38]. Unfortunately, the interfacial space charges are built when a solid electrolyte is contacted with an electrode, hindering the ion transportation and leading to the inadequate capacity and cycling performance [39].

Therefore, improvement of the ion conduction through the interface between solid-state electrolyte and electrode is the key to success. We have explored a new class of composite electrolyte aiming to enhance their ionic conductivity as well as the electrochemical/mechanical properties through constructing “ceramic framework-polymer filler” [37]. With additional ferroelectric engineering between the electrode and electrolyte, the interfacial impedance can be further reduced, resulting in the improved cyclability of the solid-state sodium-metal battery [39].

In the present work, we prepare an all-solid-state composite polymer electrolyte for the symmetric sodium-ion battery adopting NASICON-structured NVP as both cathode and anode. We also investigate the electrochemical compatibility between electrodes and the liquid as well as solid-state composite electrolyte through ab-initio molecular dynamic (AIMD) simulation. In the simulation model and the experiment tests, the cost-effective NaClO_4_ is used as the sodium salt for all the electrolytes. The AIMD simulation reveals the uncontrollable side reactions between the NVP anode and the NaClO_4_ when the liquid electrolyte is used, whereas the polymer chain in the solid-state composite electrolyte can protect NaClO_4_ from reduction on the NVP anode. To further reduce the interfacial resistance between the electrode and the composite electrolyte, the interface is tailored with the ferroelectric coating. Due to the ferroelectric effect, both the charge-transfer and SEI resistance can be effectively reduced at the interface. Based on these improvements, a symmetric NVP//NVP cell is achieved with a capacity retention of 86.4% after 650 cycles (with a reduced fading rate of 0.021% per cycle, star symbol in Fig. 1), and finally an ultra-stable all-solid-state sodium-ion battery is fabricated which can be operated stably even for 9000 cycles with a fading rate as low as 0.005% per cycle.

## Experimental Section

### Electrolyte Preparation

Porous ferroelectric-Na_3_Zr_2_Si_2_PO_12_ ceramic framework was prepared as previously reported [39]. Thin ferroelectric layers of K_0.5_Na_0.5_NbO_3_ were coated on the surface of the Na_3_Zr_2_Si_2_PO_12_ framework without affecting its NASICON crystalline structure (Fig. S1). The weight ratio of ferroelectric K_0.5_Na_0.5_NbO_3_ to Na_3_Zr_2_Si_2_PO_12_ was optimized to 2.06:100. NaClO_4_/PEO polymer precursor was synthesized through mixing NaClO_4_ (98 wt%, Sigma-Aldrich) and PEO (M_v_ 600,000 Sigma-Aldrich) at a fixed Na:EO molar ratio of 1:16. The ferroelectric-engineered composite electrolytes were fabricated by vacuum-infiltrating the PEO (NaClO_4_) into the porous framework. The infiltration process was repeated until both sides of the framework were wetted by the polymer. The composite electrolyte was further pressed into thin membranes with the thickness of 110–160 μm.

### Characterization

The morphologies of both ferroelectric-Na_3_Zr_2_Si_2_PO_12_ frameworks and the as-synthesized composite electrolytes were characterized using a field emission scanning electron microscope (HITACHI S-4300) and a transmission electron microscope (TEM, Tecnai G2 F30). The crystal structures of the samples were examined by the X-ray diffraction (XRD, Shimadzu XRD-6000). X-ray photoelectron spectroscope (XPS, Thermo Scientific ESCALAB 250Xi) were used to reveal the chemical environment of the interfacial anode/cathode interphases, after 5 cycles of charging/discharging.

The electrochemical properties of the electrolytes and battery cells were all measured using a Solartron system (1260 + 1287). The electrochemical impedance spectroscopy of the electrolytes and battery cells were measured at an AC voltage of 10 mV. The ionic conductivity of the electrolytes was calculated according to the equation: *σ* = t/RA, where *t* is the thickness, *A* is the surface area of the electrolyte, and *R* is the measured resistance from impedance spectroscopy. The electrochemical window of the electrolyte was evaluated through the linear cyclic voltammetry in the format of Na//stainless at a scanning rate of 0.5 mV s^−1^. The cyclic voltammetry measurement of the battery cells was conducted at a scanning rate of 0.1 mV s^−1^. Specifically, in the intermittent cyclic voltammetry measurements, the electrochemical impedance spectroscopy of each battery cell was evaluated under a bias of the previous voltage step. To investigate the voltage changes of the NVP anode in the 3-electrode cell (with a reference electrode of the Na metal) during battery charge/discharge, a current density of 20 mA g^−1^ was applied, with an operation voltage range from open circuit potential to 3.0 V.

### Simulation Calculations

The *ab-initio* molecular dynamics (AIMDs) was performed via the Materials Studio CASTEP module. The electronic structure was described with DFT in the local-density approximation (LDA) [40], using a plane-wave basis and ultrasoft pseudopotentials [41]. The system was canonical ensemble. The cutoff energy for plane-wave basis was set as 400 eV, and the total simulation time was 3 ps with timestep of 1 fs.

### Battery Assembly and Performance Measurements

NVP and NFFCN were synthesized as previously reported [42, 43]. The electrode slurry was prepared by mixing the active material, super P and polyethylene oxide into *N*-methyl-2-pyrrolidone in a weight ratio of 7:2:1 followed by casting onto aluminum foil and the cast electrode was finally vacuum-dried at 120 °C overnight. The mass loading of the cathode material is 0.8–1.05 mg cm^−2^. For the NVP//NVP symmetric cell, the mass ratio of NVP anode to NVP cathode is 1.02, while for the asymmetric NVP//NFFCN cell, the mass ratio of NVP to NFFCN is 1.05. All cells were assembled in glovebox (O_2_ < 1 ppm and H_2_O < 1 ppm), via cold-pressing the cathode, electrolyte, and anode into coin cells. The electrochemical performances of the cells were tested using LAND battery testing system. All batteries were measured after 1 cycle of activation at 10 mA g^−1^.

## Results and Discussion

**Fig. 2:**
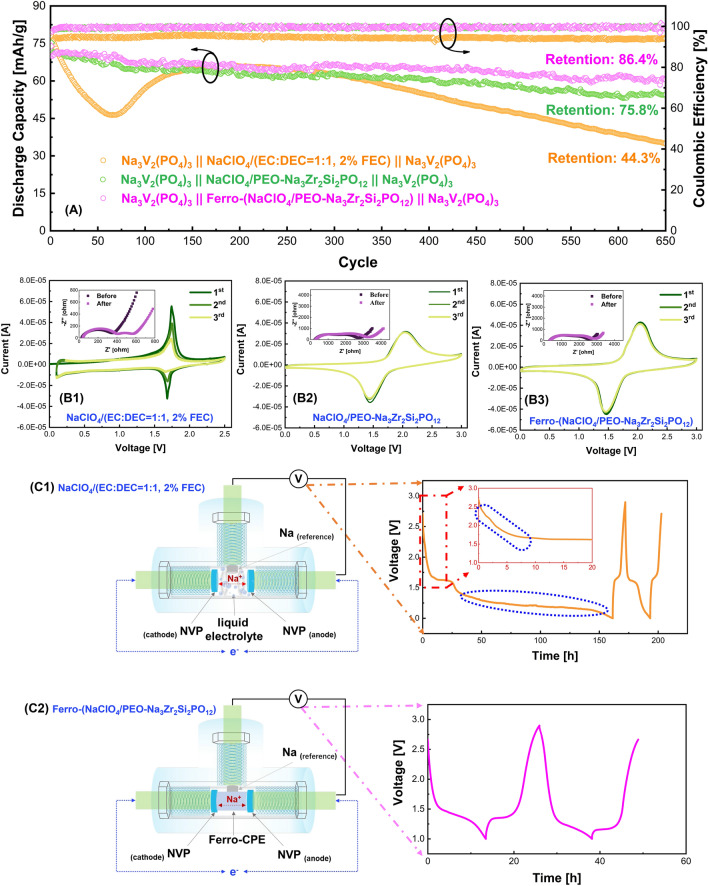
**A** Cycling performance of the symmetrical NVP//NVP cells using the commercial liquid electrolyte and all-solid-state non-/ferroelectric-engineered composite electrolyte (0.5C, 1C = 118 mAh g^−1^), CV profiles of the NVP//NVP symmetric cells with **B1** commercial liquid electrolyte, **B2** non-engineered composite electrolyte, **B3** ferroelectric-engineered composite electrolyte (Insets are the Nyquist plots of the cells before and after CV measurements), 3-electrode cell configuration and the voltage profile of the NVP anode in the cell using **C1** commercial liquid electrolyte and **C2** all-solid-state ferroelectric-engineered composite electrolyte. All measurements are conducted at room temperature

The all-solid-state ferroelectric-engineered composite electrolyte which was prepared by fully filling NaClO_4_/PEO into the 3D-porous ferroelectric-engineered Na_3_Zr_2_Si_2_PO_12_ ceramic frameworks enables not only high flexibility but also high ionic conductivity (Figs. S1 and S2). Compared with the bare composite electrolyte termed as non-engineered composite electrolyte, the ferroelectric tailoring widened the electrochemical window of the resultant electrolyte. Moreover, symmetric solid-state sodium-ion battery demonstrated stable cyclability as shown in Fig. 2A. The capacity retention is as high as 86.4% after 650 cycles with only a fading rate of 0.021% per cycle for the NVP//NVP cell using the all-solid-state ferroelectric-engineered composite electrolyte, whereas the cell using the commercial liquid electrolyte fades about 0.086% per cycle, 4 times faster than the ferroelectric-engineered cell. Although the symmetric cell using the conventional non-engineered composite electrolyte achieves improved cyclic stability with a capacity retention of 75.8% over 650 cycles in comparison with the liquid cell, its fading rate (0.037% per cycle) is still unsatisfied and much higher than the ferroelectric-engineered symmetric cell.

### Electrolyte–Electrode Interfacial Stability in Symmetric NVP//NVP Cells

To study the role of the electrolyte on the electrochemical performance of the symmetrical cells, the cyclic voltammetry (CV) measurements of the symmetric NVP//NVP cells using various electrolytes were conducted. Since NVP possesses two pairs of redox reactions, one of which is at ~ 3.4 V (Na/Na^+^) and another at ~ 1.7 V (Na/Na^+^), it can be used as an anode as well as the cathode, with a voltage of ~ 1.7 V in a symmetric cell [7]. During the CV measurement, however, two irreversible oxidic peaks at ~ 1.0 and ~ 1.5 V were observed in the symmetric cell using the commercial liquid electrolyte containing NaClO_4_ as the sodium salt, as shown in Fig. 2(B1), agreeing with the differential capacity analysis derived from the long-term cycling data (Fig. S3). Moreover, the intensity of the 1.7 V-redox peaks decreases with charge/discharge cycles, which is associated with the increased resistance in the cell, indicating some unexpected parasitic reactions happening. To avoid these parasitic reactions, we tried to narrow the operation voltage of the cell using the liquid electrolyte down to 1.5–2 V from previous 0.8–2.5 V. Unfortunately, the cell could still not perform well (Fig. S4). At a low operating current density, the capacity almost faded to 0 just within 10 cycles.

In contrast, there was no irreversible oxidic reaction in the symmetric solid-state cells using the non-engineered (Fig. 2(B2)) or ferroelectric-engineered composite electrolyte (Fig. 2(B3)), consistent with the differential capacity analysis in Fig. S3. Furthermore, the total resistance of the all-solid-state cells only slightly increased after the CV measurement, especially in the NVP || ferro-(NaClO_4_/PEO-Na_3_Zr_2_Si_2_PO_12_) || NVP symmetric cell with almost no increase. The analyses on the symmetric cells revealed that the all-solid-state composite electrolytes are more compatible with the NVP electrodes than the commercial liquid electrolytes.

It has been shown that the commercial liquid electrolyte, such as NaClO_4_/(EC-DEC, FEC as additive), is electrochemical stable with the NASICON-structured cathodes [42, 44]. The CV profiles shown in Fig. S5(A) also confirm that the liquid electrolyte is electrochemical stable with the NVP cathode (based on V^+3/+4^ redox reaction). However, the poor compatibility between the NVP anode (based on V^+3/+2^) and the liquid electrolyte is noted, as shown in the CV diagrams in Fig. S5(B). Further study of the differential capacity profile in Fig. S6 shows the irreversible reduction in NVP anode between 1.0 and 1.4 V (Na/Na^+^) when the liquid electrolyte was used, whereas no obvious side reaction was observed between the NVP anode and the ferroelectric-engineered composite electrolyte.

To explore the electrochemical reaction when NVP is used as an anode in the NVP//NVP symmetric cell, we carefully recorded the voltage variation of the NVP anode using a 3-electrode cell in which an additional Na metal was used as the reference electrode in a T-shape Swagelok cell. As observed in the cell using the liquid electrolyte in Fig. 2(C1), during discharge the NVP anode firstly experienced irreversible reduction from the beginning till 1.6 V (Na/Na^+^), followed by the partially reversible reaction at ~ 1.6 V (Na/Na^+^) and then a serious irreversible reaction from 1.4 to 1.0 V (Vs. Na/Na^+^). The irreversible reactions are highlighted in blue circle. On the other hand, almost no irreversible reaction was observed in the NVP cathode (Fig. S7). Therefore, the quick capacity fade in the symmetric NVP//NVP cells is believed mainly associated to the anode.

Two possible causes which can be speculated at this stage of investigation are: (a) serious irreversible side reactions between the liquid electrolyte and NVP anode in the first cycle (Figs. 2(C1) and S4), and (b) continuous consumption of Na^+^ between 1.4 and 1.0 V (Na/Na^+^) in the following cycles which can be clearly reflected by Fig. S6. Noted that the open circuit potential for the NVP anode is ~ 2.7 V (Na/Na^+^) and its effective redox potential (V^+3/+2^, with one Na^+^ occupying the 18e site [45]) is ~ 1.6 V Vs. Na/Na^+^, thus no matter how narrow the operation voltage range is set for the symmetric cell, the irreversible reduction of the NVP anode is unavoidable when using the liquid electrolyte, as indicated by the irreversible reduction on NVP anode from 2.7 to 1.6 V (Na/Na^+^) in Fig. 2(C1). On the contrary, only slight irreversible reaction was observed from the all-solid-state cell (Fig. 2(C2)), indicating that the all-solid-state ferroelectric-engineered composite electrolyte possesses high electrochemical compatibility with the NVP anode, which is the vital issue for a cyclic stable NVP//NVP symmetric cell.

**Fig. 3:**
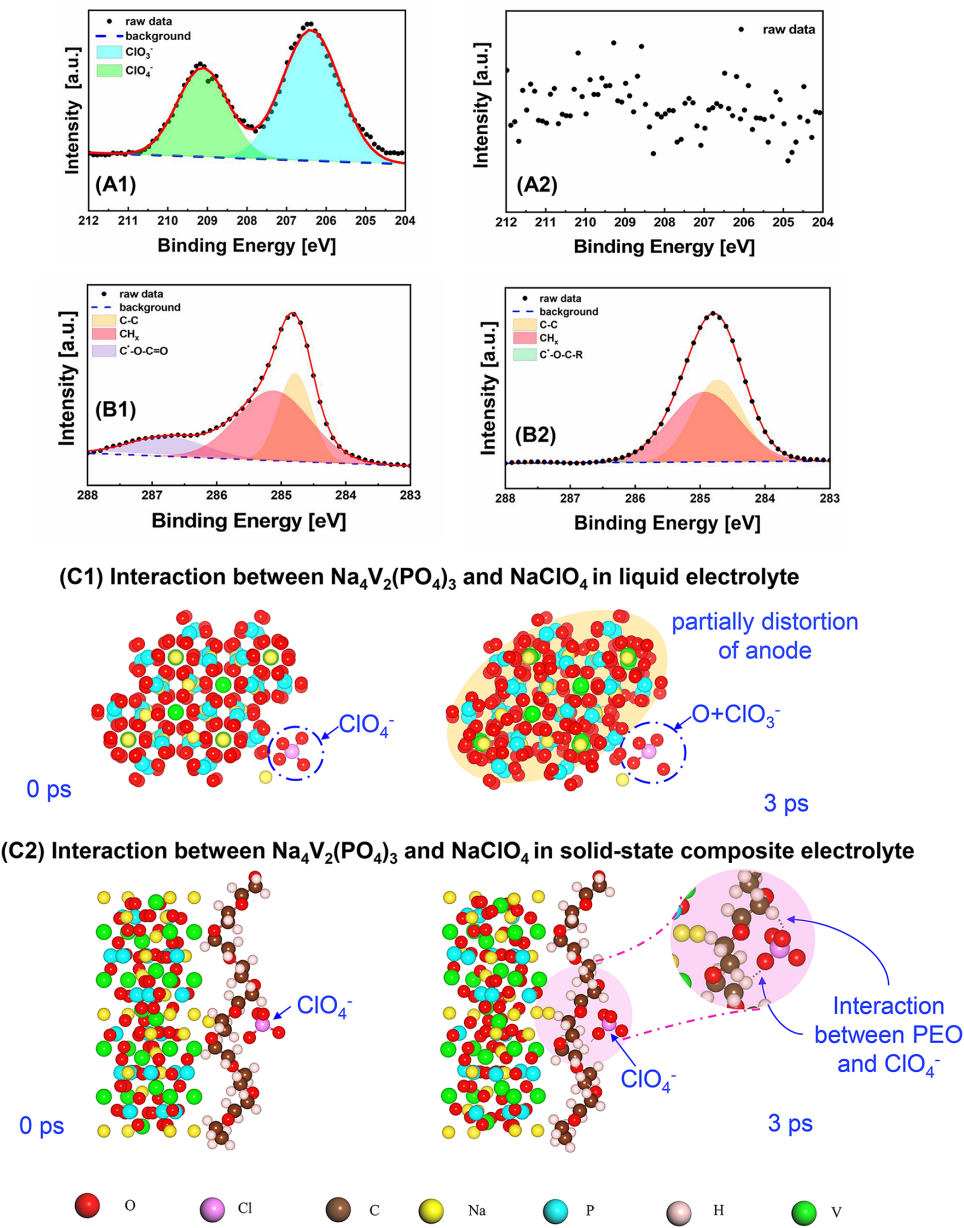
XPS analyses of the Cl and C signal at the interfacial anode disassembled from the NVP//NVP cell using **A1**, **B1** commercial liquid electrolyte and **A2**, **B2** all-solid-state ferroelectric-engineered composite electrolyte, **C1** simulated interaction between the NVP anode and NaClO_4_ in liquid electrolyte, **C2** simulated interaction between the NVP anode and NaClO_4_ in the all-solid-state composite electrolyte

To further understand the cause of the irreversibility of the NVP anode, we used the XPS to investigate the possible SEI components on the NVP anodes which were disassembled from the liquid and solid-state cells, respectively. As illustrated in Fig. 3(A1), (A2), ClO_3_^−^, and ClO_4_^−^ are clearly observed on the surface of the NVP anode when liquid electrolyte was used, indicating the reduction of ClO_4_^−^, whereas the ClO_3_^−^/ClO_4_^−^ signal is negligible on the surface of NVP anode when the ferroelectric-engineered composite electrolyte was used. In addition, the Cl signal is absent on the surface of NVP cathode regardless liquid electrolyte or ferroelectric-engineered composite electrolyte being used (Fig. S8). Considering the ClO_4_^−^ has higher oxidizability than the NVP anode, it could grab the electrons from the anode side, being reduced to ClO_3_^−^ and forming SEI on the anode, which explains the irreversible reduction between 2.7 and 1.6 V (Vs. Na/Na^+^) in the liquid cell (Fig. 2(C1)). At 1.6 V (Na/Na^+^), the Na_3_V_2_(PO_4_)_3_ anode is fully reduced into Na_4_V_2_(PO_4_)_3_, and therefore a voltage plateau is observed. After the conversion of Na_4_V_2_(PO_4_)_3_, a long irreversible reduction is observed below 1.3 V (Na/Na^+^) (Fig. 2(C1)), which is most probably due to the decomposition of the solvent in the liquid electrolyte (Fig. S9). In the XPS analyses of the C element (Fig. 3(B1)), C–O–C=O peak appears together with the C–C/C–H peak on the NVP anode when using the liquid electrolyte. Despite the C–C/C–H that might also come from the carbon black or polymer binder within the anode, the appearance of C–O–C=O peak supports the speculation that the ester solvent in the liquid electroltye is decomposed, even though the protective NaF SEI is already formed on the anode (Fig. S10). On the contrary, C–C, C–H, and a tiny C–O–C–R are observed on the NVP anode when using the all-solid-state composite electroyte. In the solid-state cell, these peaks might be either attributed to the additives within the anode (e.g., carbon black, polymer binder) or the PEO within the composite electrolyte. However, the absence of Cl signal in the solid-state cell indicates that the all-solid-state composite electrolyte could improve the stability between the NaClO_4_ salt and the NVP anode, consistent with the electrochemical investigation in Fig. 2(C2).

The electrochemical compatibility between the NVP anode and the liquid/solid-state electrolyte has been further investigated through the *ab-initio* molecular dynamic (AIMD) simulation. The stability between the anode Na_4_V_2_(PO_4_)_3_ and the sodium salt (represented by the pair of Na^+^ and ClO_4_^−^ as their dissolved states in the liquid electrolyte) is very poor. As shown in Fig. 3(C1), ClO_4_^−^ is reduced to ClO_3_^−^ just after 3 ps, consistent with the observation from XPS results. Together with the reduction of the ClO_4_^−^, the lattice structure of the NVP anode is partially distorted. Considering the short period (3 ps) the simulation could apply, serious lattice distortion or even collapse of the NVP anode could be speculated after the long-time reaction with the liquid electrolyte, probably resulting in the capacity fading. However, for the all-solid-state composite electrolyte, the sodium salt (NaClO_4_) is dissolved into the PEO polymer. Na^+^ could migrate with the segmental movements of the polymer chain and insert into/extract from the cathode/anode. The ClO_4_^−^ is on the other hand effectively prevented from the electrode surface by the PEO chain via the weak interaction, as shown in Figs. 3(C2) and S11. In addition, hydrogen bonds could be formed between the H from the end group of PEO and the O of ClO_4_^−^. Then, ClO_4_^−^ could not grab the electrons from the NVP anode directly. After 3 ps, no clear reaction could be observed between the anode Na_4_V_2_(PO_4_)_3_ and ClO_4_^−^. Furthermore, the stability between the NVP anode and the other components of the ferroelectric-engineered composite electrolyte was also simulated. As shown in Fig. S12(A)–(C), there is almost no side reaction between the NVP anode and the ceramic/polymer components (including the Na_3_Zr_2_Si_2_PO_12_, ferroelectric K_0.5_Na_0.5_NbO_3_ and the PEO polymer) in the composite electrolyte. All these results indicate the improved interfacial stability between the NVP anodes and the solid-state composite electrolytes, in comparison with the commercial liquid electrolyte. However, it should be noted that the appearance of water molecules would break the stability between the NVP anode and the PEO polymer chain. Figure S12(D) reveals that the water molecules can destroy the polymer chain, leading to the decomposition of NVP anode. Thus, to improve the cycling performance of the solid-state symmetric NVP//NVP cells, moisture should be carefully avoided from the fabrication environment.

### Electrode–Electrolyte Interfacial Resistance in All-Solid-State NVP//NVP Cells

**Fig. 4:**
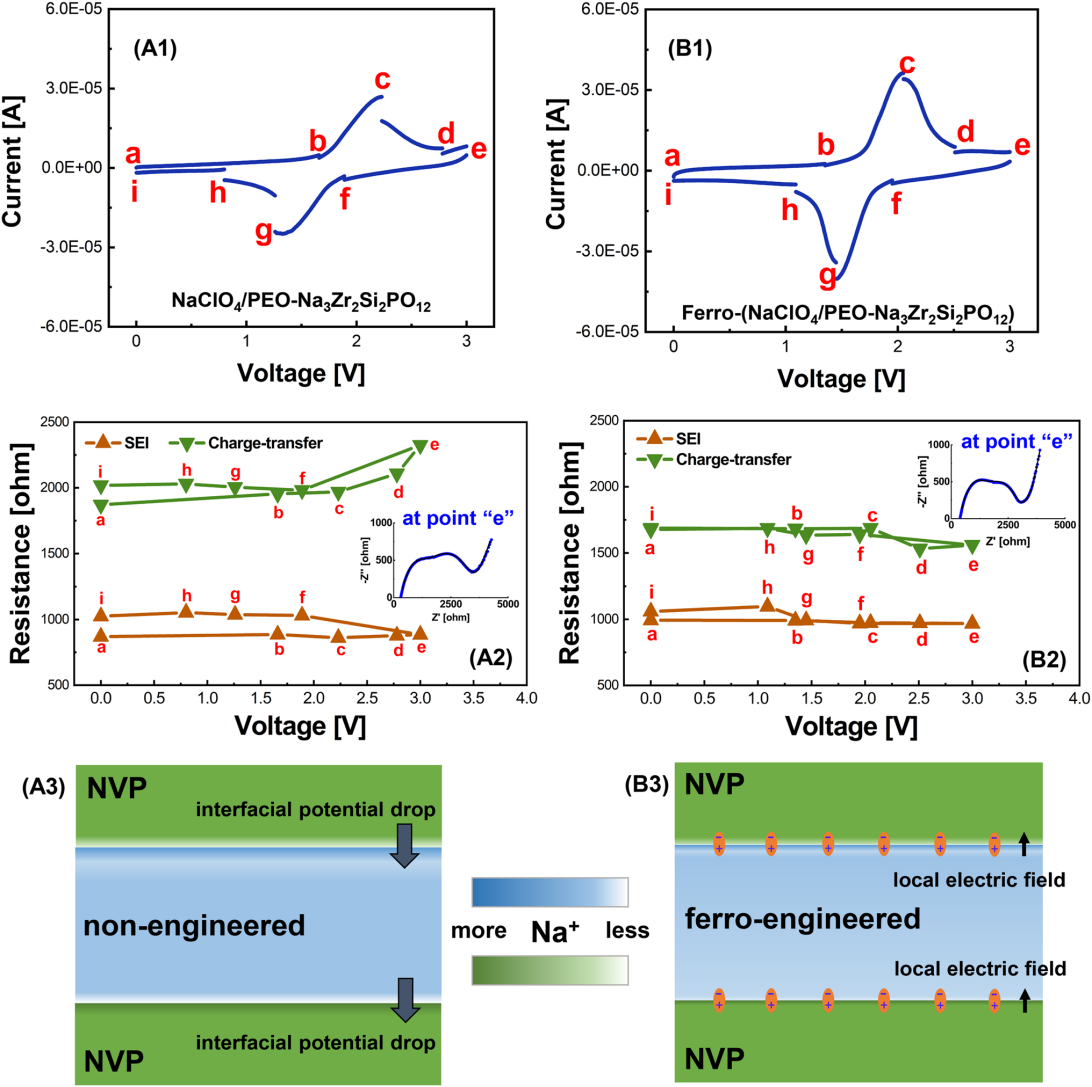
Intermittent CV profiles, interfacial resistances, and the schematic drawing of the interfacial ion distribution in all-solid-state NVP//NVP symmetric cells using **A1**, **A2**, **A3** non-engineered composite electrolyte and **B1**, **B2**, **B3** ferroelectric-engineered composite electrolyte. Insets are the Nyquist plots of the cells at point e. All measurements are conducted at room temperature

Compared with the cells using liquid electrolyte, the interfacial stability of the electrode can be greatly improved using the all-solid-state composite electrolytes. In addition to the interfacial stability, the electrode–electrolyte interfacial impedance is another critical factor. To investigate the change of interfacial impedance of the all-solid-state cells, the intermittent CV measurements were carried out. The electrochemical impedance of the cells was recorded at various intermittent stage of charge and discharge, labeled from “*a”* to “*i”* in Fig. 4(A1), (B1). For the non-engineered composite electrolyte, there is a big current change, whereas for the ferroelectric-engineered composite electrolyte, the current drop/increase at the anodic/cathodic sweeping intermittent stage is neglectable. This observation implies that the ferroelectric-engineered composite electrolyte possesses much better kinetics for Na-ion transportation.

From the Nyquist plots (Fig. S13), two obvious semicircles could be observed for both cells at various voltages: one at middle frequency and the other one at low frequency. The semicircle at middle frequency is attributed to the SEI resistance with the characteristic frequency of 10^3^–10^4^ Hz, whereas the semicircle at low-frequency represents the charge-transfer resistance with the characteristic frequency of 10–100 Hz [46]. Since it is a symmetric cell, the characteristic frequency of charge transfer for both anode and cathode could be considered the same. As demonstrated in Fig. 4(A2), (B2), the charge-transfer resistance is the major issue resisting the ion conduction. The resistance is higher in charge and lower in discharge for the cell using the non-engineered composite electrolyte. The resistance becomes the largest when the cell is at the highest voltage of 3.0 V (point “*e*”). As known, the potential difference between the electrode and electrolyte would cause the space-charge accumulating at the interface and increase the energy barrier for ion migration [47, 48]. The largest charge-transfer resistance for the non-engineered cell could be explained by the excessive space-charge accumulation when the cell is under the highest voltage. As reported in our recent work [39] and also illustrated in the schematics of Fig. 4(A3), (B3), the interfacial potential between the electrode and the electrolyte would be able to polarize the ferroelectric domains and generate an opposite electric field at the interfaces, by rotating the ferroelectric dipoles. This additional local electric field can therefore redistribute the interfacial ions and suppress their accumulation at the electrode–electrolyte interfaces. Therefore, reduced charge-transfer resistance could be achieved in the ferroelectric-engineered cell. Moreover, under the high voltage at point “*d*” and “*e*,” even slightly decreased charge-transfer resistances are demonstrated in the ferroelectric-engineered cell, revealing that the interfacial ion migration could be further facilitated via the ferroelectric effect.

Different from the charge-transfer resistance, the SEI resistance in the non-engineered cell (Fig. 4(A2)) remains stable during charge but increases in the following discharge, however, always larger than that in the ferroelectric-engineered cell during the full charge/discharge period. Interestingly, the resistance could remain stable in the ferroelectric-engineered cell, except at the end of discharge at about 1.1 and 0 V (Fig. 4(B2)). According to DFT calculation, the NVP anode would experience a large volume change of ~ 5.24% (Fig. S14) during charge/discharge. The integrity including partial delamination and crack of the SEI formed on the NVP anode might be destroyed due to the large volume change. As a consequence, new SEI may form, resulting in increased SEI resistance of the non-engineered cell. Thanks to the piezoelectric effect of the ferroelectric phases, the stress induced by the volume change of NVP anode can be relieved. The mechanical energy could be converted into electrical energy via the piezoelectric effect or consumed by the stress-induced phase transition in ferroelectrics. Therefore, stable SEI resistance could be maintained in the cell using the ferroelectric-engineered composite electrolyte. However, the electromechanical coupling coefficient of the ferroelectrics which indicates the energy conversion efficiency would be reduced under lower applied voltage [49]. The SEI resistance therefore increases slightly in the ferroelectric-engineered cell when the cell is discharged below 1.1 V. Even so, the increase of the SEI resistance in the ferroelectric-engineered cell is as low as about 13.3% compared to ~ 22.2% of the cell using the non-engineered composite electrolyte, when the cells are under low voltages (at points of “*h*” and “*i*”).

### Electrochemical Performance of All-Solid-State NVP//NFFCN Cells

**Fig. 5:**
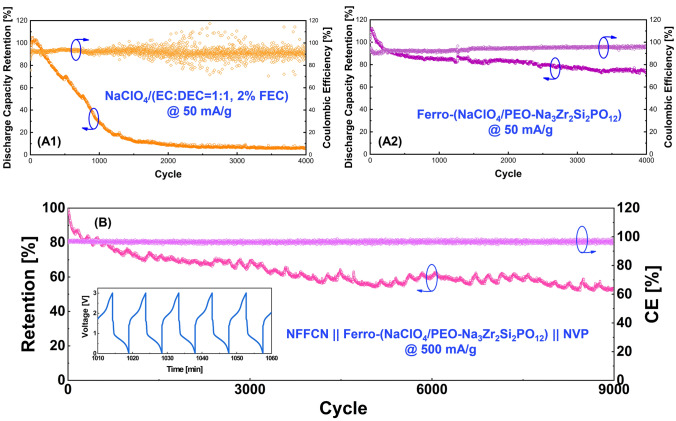
Cycling performance of the NVP//NFFCN asymmetric cells using **A1** commercial liquid electrolyte and **A2** all-solid-state ferroelectric-engineered composite electrolyte (current density of 50 mA/g, room temperature), **B** Long-term cycling performance of the NFFCN || Ferro-(NaClO_4_/PEO-Na_3_Zr_2_Si_2_PO_12_) || NVP cell under 500 mA g^−1^ at room temperature. Inset indicates the cell could complete one charge/discharge cycle within 10 min

To confirm the above analyses, we further fabricated asymmetric cell using Prussian-blue Na_*x*_Fe_*y*_Fe(CN)_6−*z*_·nH_2_O (NFFCN) as cathode, NVP as anode and the ferroelectric-engineered composite electrolyte. For a comparison, a cell using commercial liquid electrolytes was also fabricated. Figure 5 demonstrates the long-term cyclability of the cells. Obviously, a superior electrochemical performance is achieved for the all-solid-state battery, namely using the ferroelectric-engineered composite electrolyte, with a discharge capacity retention of 73.1% after 4000 cycles at a current density of 50 mA g^−1^ (Fig. 5(A2)), and a retention of 53.3% after 9000 cycles at a high current density of 500 mA g^−1^ (as shown in Fig. 5(B)). The capacity fading rate of the cell is as low as ~ 0.005% per cycle, demonstrating a breakthrough in the field of the all-solid-state sodium-ion batteries. On the other hand, the cell using the liquid electrolyte shows a steep capacity fading after only 1000 cycles at the small current density of 50 mA g^−1^ (Fig. 5(A1)).

It should be mentioned that the present ferroelectric-engineered composite electrolyte could still not wet the thick electrodes well, even though its intrinsic flexibility already improves the electrode–electrolyte contact. If we could design an integrated electrode/electrolyte structure [50] and enhance the ionic/electronic conduction within the electrodes, especially within the NVP anode, an attractive energy–density as well as further improved cyclic performance would be obtained in the all-solid-state sodium-ion batteries.

## Conclusions

In this work, we have developed an all-solid-state ferroelectric-engineered composite electrolyte to improve the electrode–electrolyte interfacial stability as well as the interfacial ion conduction in the sodium-ion battery using the NASICON-structured Na_3_V_2_(PO_4_)_3_ as both cathodes and anodes. The all-solid-sate Na_3_V_2_(PO_4_)_3_ || ferro-(NaClO_4_/PEO-Na_3_Zr_2_Si_2_PO_12_) || Na_3_V_2_(PO_4_)_3_ symmetric cell demonstrated a discharge capacity retention of 86.4% after 650 cycles, obviously superior over the cells using the commercial liquid electrolytes or the conventional solid-state electrolytes. Moreover, this ferroelectric-engineered composite electrolyte could serve other solid-state sodium-ion batteries. Ultra-stable long-term cycling performance was achieved in the all-solid-sate Na_*x*_Fe_*y*_Fe(CN)_6−*z*_·nH_2_O || ferro-(NaClO_4_/PEO-Na_3_Zr_2_Si_2_PO_12_) || Na_3_V_2_(PO_4_)_3_ cell under a high current density of 500 mA g^−1^, with the discharge capacity retention of 53.3% after 9000 cycles, namely the fading rate was only ~ 0.005% per cycle. Our work provides a new path for designing the practical large-scale stationary batteries with long life, high safety and attractive cycling performance.

## Supplementary Information

Below is the link to the electronic supplementary material.

Supplementary file1 (PDF 2043 KB)
