# Supplementary material for: Ultra-Stable Sodium-Ion Battery Enabled by All-Solid-State Ferroelectric-Engineered Composite Electrolytes
Source: Nanomicro Lett. 2024 Jul 25;16:254. doi: 10.1007/s40820-024-01474-6 (PMC11272761; doi:10.1007/s40820-024-01474-6)
Supplement: Supplementary file 1 — Supplementary file1 (PDF 2043 KB) [file 40820_2024_1474_MOESM1_ESM.pdf]

Supporting Information for

Ultra-Stable Sodium-Ion Battery Enabled by All-Solid-State Ferroelectric-Engineered Composite Electrolytes

Yumei Wang[1,2,#,] *, Zhongting Wang[3,#], Xiaoyu Xu[1,2,4], Sam Jin An Oh[4,] Jianguo Sun[4], Feng Zheng[4], Xiao Lu[4], Chaohe Xu[1,]*, Binggong Yan[5], Guangsheng Huang[3], Li Lu[4,]*

[1]College of Aerospace Engineering, Chongqing University, Chongqing 400044, P. R. China

[2]National University of Singapore (Chongqing) Research Institute, Chongqing 401123, P. R. China,

[3]College of Materials Science and Engineering, Chongqing University, Chongqing 400044, P. R. China

[4]Department of Mechanical Engineering, National University of Singapore, 9 Engineering Drive 1, Singapore 117575, Singapore

[5]Fujian Key Laboratory of Special Energy Manufacturing, Xiamen Key Laboratory of Digital Vision Measurement, Huaqiao University, Xiamen, P. R. China

[#] Yumei Wang and Zhongting Wang contributed equally to this work.

*Corresponding authors. E-mail: yumei_emma@qq.com (Yumei Wang); xche@cqu.edu.cn (Chaohe Xu); luli@nus.edu.sg (Li Lu)

Supplementary Figures

As shown in Fig. S1, thin ferroelectric layers of $K_{0.5}Na_{0.5}NbO_3$ are successfully coated on the surface of the $Na_3Zr_2Si_2PO_{12}$ framework without affecting its NASICON crystalline structure, since no impurity phases could be observed from the XRD analyses. The 3D interconnected pores have been maintained in the ferroelectric-engineered framework (termed as ferroelecitric-$Na_3Zr_2Si_2PO_{12}$), as presented in the morphology image. Then, the polymer filler of $NaClO_4$/PEO is fully filled into the pores of the framework. In this way, thin and highly flexible ferroelectric-engineered composite electrolyte is prepared, within which interconnected and continuous ceramic-polymer interfaces are formed. The ion could migrate fast on these continuous ceramic-polymer interfacial pathways.

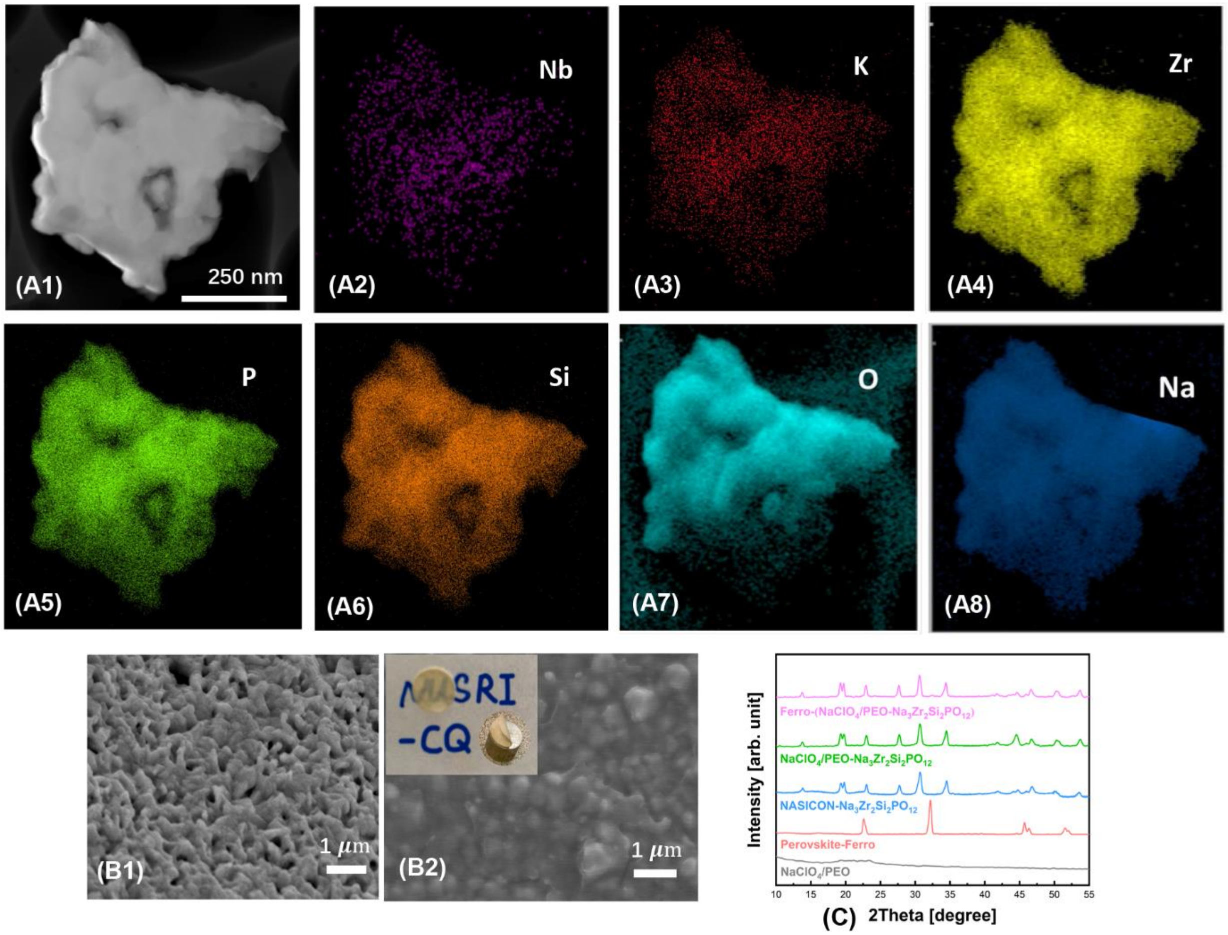

Fig. S1 (**A1**) HAADF image, (**A2**) Nb mapping, (**A3**) K mapping, (**A4**) Zr mapping, (**A5**) P mapping, (**A6**) Si mapping, (**A7**) O mapping and (**A8**) Na mapping of the porous ferroelectric-$Na_3Zr_2Si_2PO_{12}$ framework, morphology of the (**B1**) porous ferroelectric-$Na_3Zr_2Si_2PO_{12}$ framework and (**B2**) $NaClO_4$/PEO fully filled ferroelectric-$Na_3Zr_2Si_2PO_{12}$ (inset is the digital photo of the thin and flexible composite electrolyte), (**C**) XRD patterns of $NaClO_4$/PEO polymer membrane, Perovskite-ferroelectric ceramics, NASICON-$Na_3Zr_2Si_2PO_{12}$, non- and ferroelectric-engineered $NaClO_4$/PEO-$Na_3Zr_2Si_2PO_{12}$ composite electrolytes

In Fig. S2, satisfied ionic conductivity is achieved in the all-solid-state ferroelectric-engineered composite electrolyte. Although the ferroelectric itself could not conduct Na^+, the ceramic/polymer interfaces provide a fast ion conduction pathway for the Na^+ within the ferroelectric-engineered composite electrolyte. At 30 ℃, the ionic conductivity is 8.96×10^{-5} S/cm and 1.6×10^{-4} S/cm for the ferroelectric-engineered and non-engineered composite electrolyte, respectively. Furthermore, the electrochemical window is broadened to 5.0 V (Vs. Na/Na^+) in the ferroelectric-engineered composite electrolyte, compared with the non-engineered electrolyte.

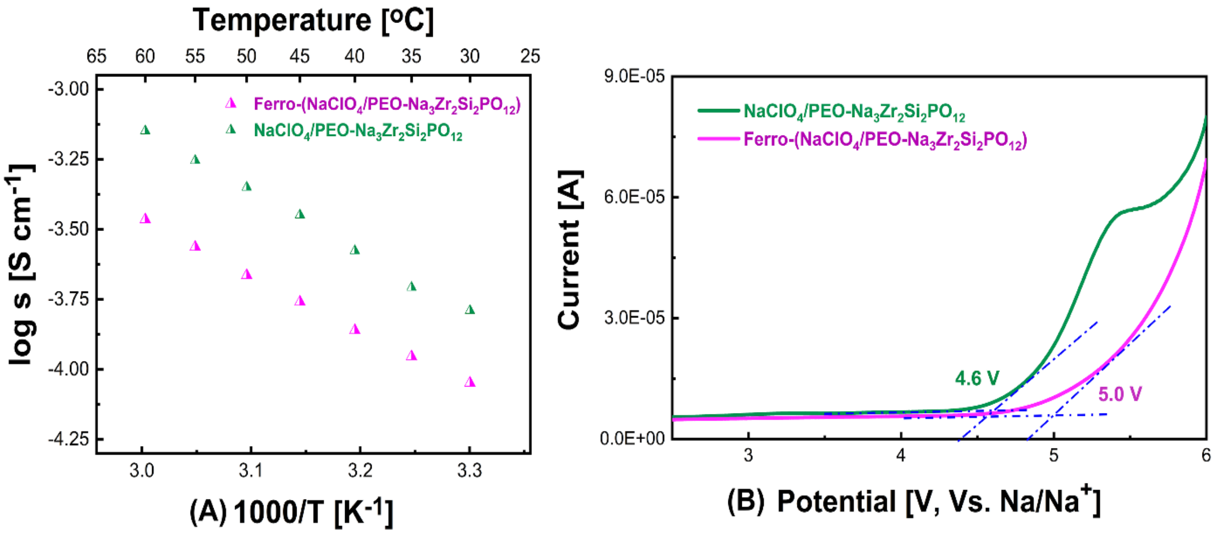

Fig. S2 (**A**) Ionic conductivity and (**B**) electrochemical window of the non- and ferroelectric-engineered $NaClO_4$/PEO-$Na_3Zr_2Si_2PO_{12}$ composite electrolytes (measured at room temperature)

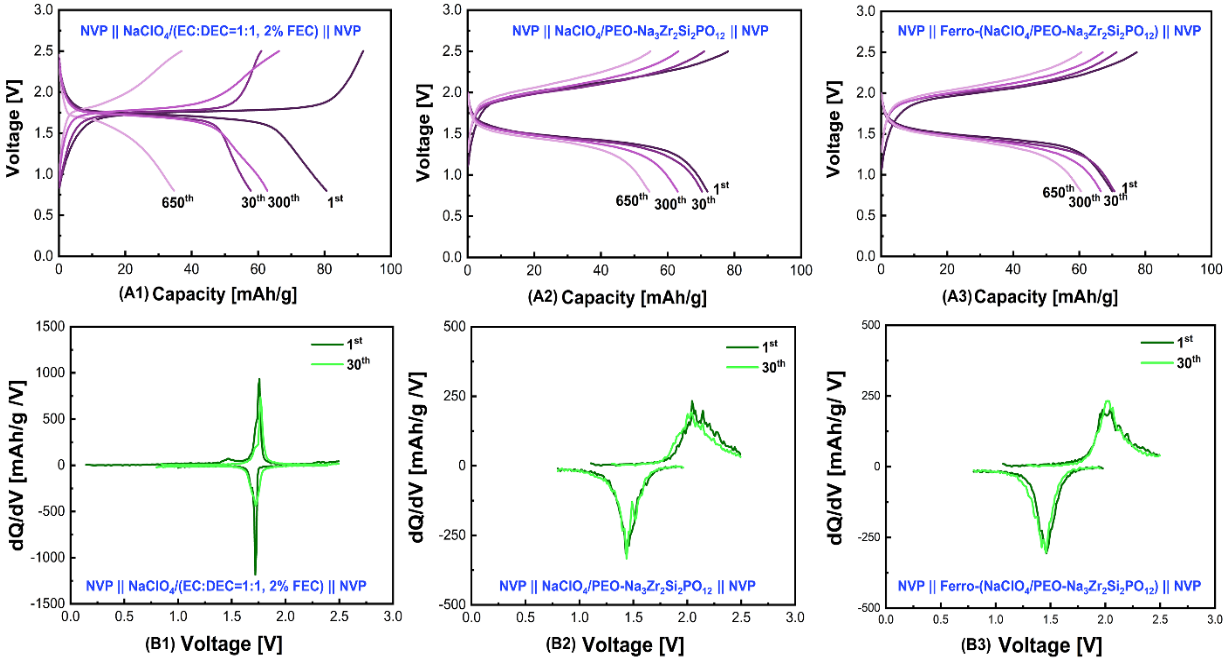

Fig. S3 Charge-discharge voltage profiles and the differential capacity profiles of the NVP//NVP symmetric cells using (**A1**) (**B1**) commercial liquid electrolyte, (**A2**) (**B2**) non-engineered composite electrolyte, and (**A3**) (**B3**) ferroelectric-engineered composite electrolyte. All measurements are conducted at room temperature

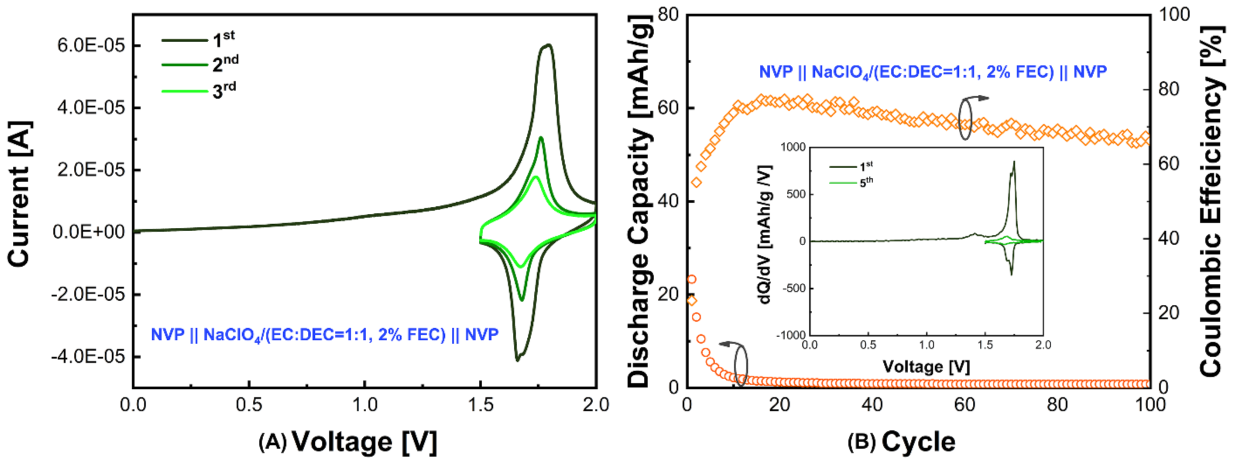

Fig. S4 (**A**) Cyclic voltammetry profile and (**B**) cycling performance of the NVP//NVP symmetric cell using liquid electrolyte (operation voltage range: 1.5-2.0 V, current density: 0.1C, 1C=118 mAh g^{-1}). Inset is the differential capacity plot. All measurements are conducted at room temperature

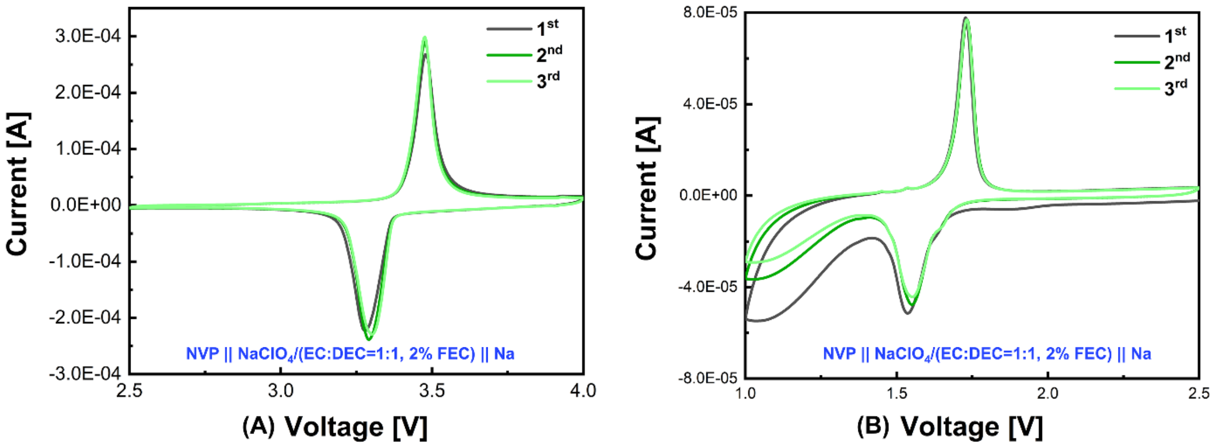

Fig. S5 Cyclic voltammetry profiles of (A) Na//NVP cell in the voltage range of 2.5-4.0 V, (B) Na//NVP cell in the voltage range of 1.0-2.5 V. All measurements are conducted at room temperature

As shown in Fig. S6, the NVP anode (based on $V^{+3/+2}$) delivers abnormally high discharge capacity when using the commercial liquid electrolyte, especially in the first 5 cycles, even higher than the theoretical value (55 mAh g^{-1}). However, this high capacity could not be maintained and keeps decreasing in the following cycles. Moreover, the coulombic efficiency of the cell is always less than 100% in the measured cycles, when using the liquid electrolyte. Thus, we believe the irreversible side reactions between the NVP anode and the liquid electrolyte contribute to the additional capacity. However, the continuous side reactions between 1.4 V and 1.0 V (Na/Na^+) always consumes Na^+, as the capacity keeps decreasing in the measured cycles, even though the Na metal in the half cell already provides sufficient Na^+. In contrast, the good cyclability could be achieved in the cell when the all-solid-state ferroelectric-engineered composite electrolyte is used,

indicating the good compatibility between the NVP anode and the all-solid-state electrolyte (Fig. S6(A), (B2)).

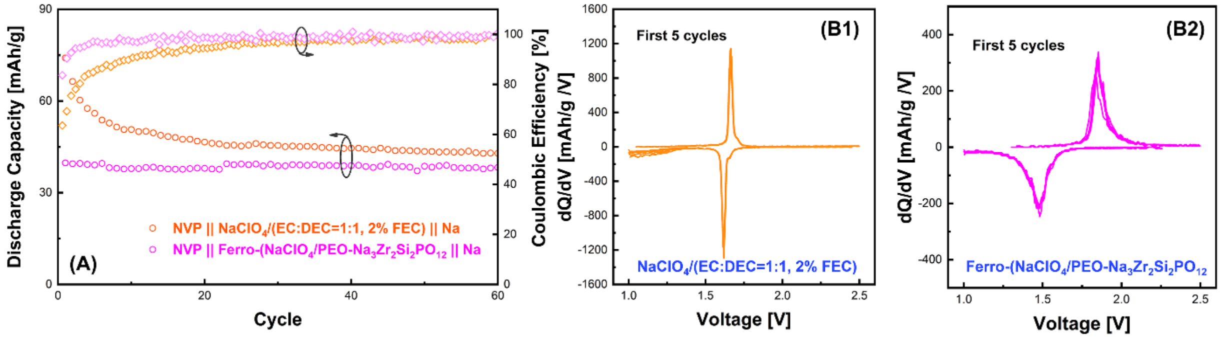

Fig. S6 (**A**) Cycling performance, (**B2**) and (**B3**) differential capacity (dQ/dV) plots of the Na//NVP cells using the commercial liquid electrolyte and all-solid-state ferroelectric-engineered composite electrolyte, respectively (operation voltage range: 1.0-2.5 V, current density: 0.1C, 1C=118 mAh g^{-1}.)

In Fig. S7, except the reversible redox reaction of $V^{+3/+4}$ at ~3.4V (Na/Na^+), very tiny irreversible oxidation could also be observed in the cathode NVP from ~3.7 V to 4.0 V Vs. Na/Na^+. This irreversible oxidation could be attributed to the stable CEI formation and did not affect the electrochemical performance of the NVP cathode.

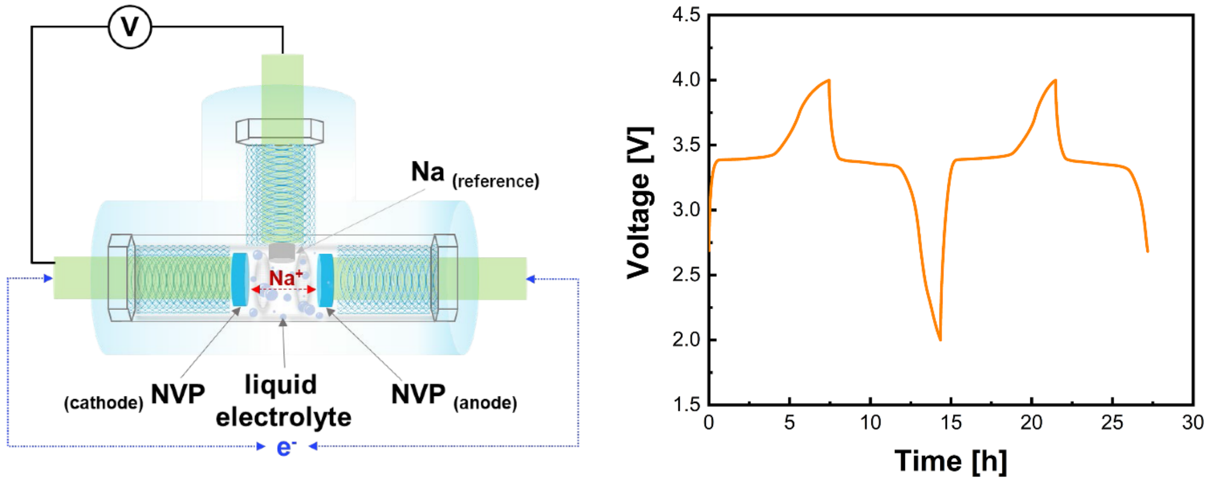

Fig. S7 3-electrode cell configuration and the voltage profile of the NVP cathode in the cell using the commercial liquid electrolyte. The measurement is conducted at room temperature

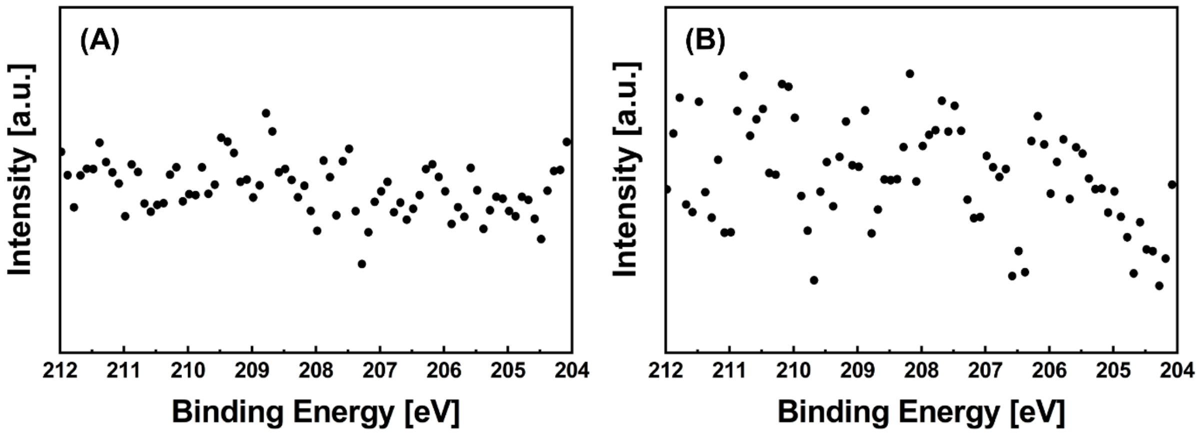

Fig. S8 XPS analyses of the interfacial cathode disassembled from the NVP//NVP cell using (**A**) commercial liquid electrolyte and (**B**) all-solid-state ferroelectric-engineered composite electrolyte

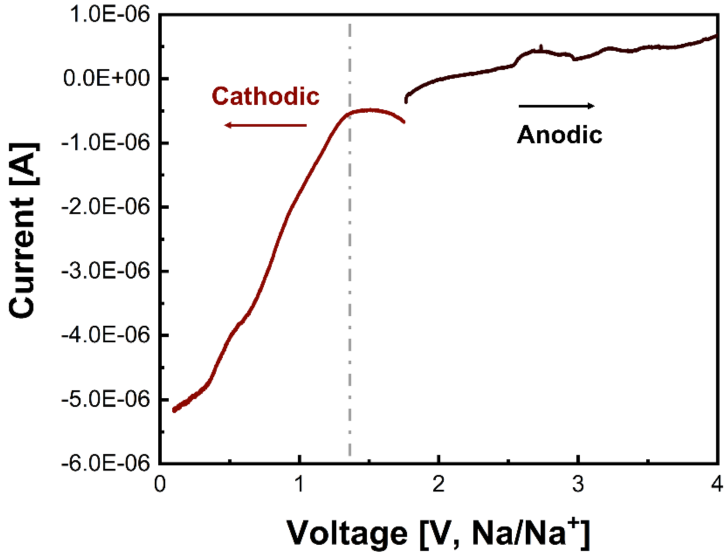

Fig. S9 LSV curve of the stainless steel || commercial liquid electrolyte || Na cell

Figure S10 shows the XPS analyses of the anodes that were disassembled from the liquid and solid-state symmetric cells, respectively. The appearance of Na, P and V peaks is mainly attributed to the active material of NVP within the anode. With the addition of FEC in the liquid electrolyte, NaF has been successfully formed at the anode interface. Together with NaF, some organic SEI was formed, as indicated by the Cl-O/C-O peak in Fig. S10 and the C-O-C=O peak in Fig. 3. In the solid-state cell, we could not collect the signal of Si/Zr/K/Nb at the anode interface, probably because the ceramic framework within the electrolyte has been fully removed during the battery disassembly. The appearance of C-O/Cl-O peak in Fig. S10 and the C-C/C-H peak in Fig. 3 could either come from the polymer within the composite electrolyte or the binder within the anode.

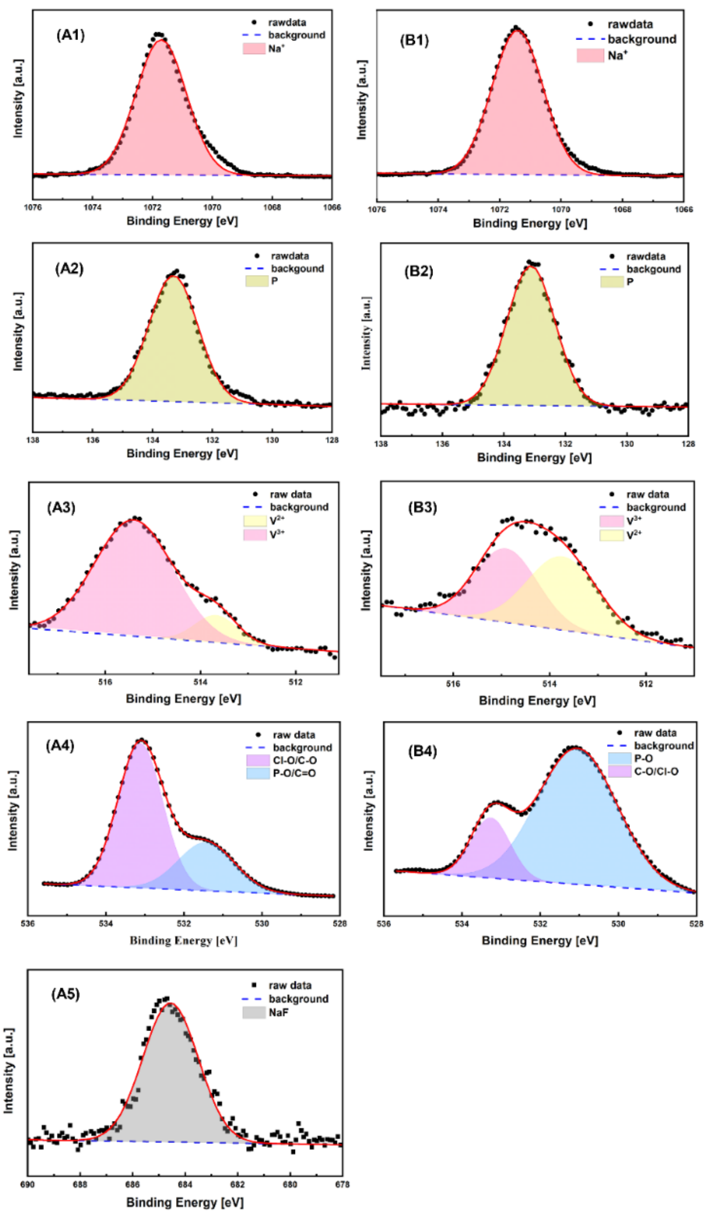

Fig. S10 XPS analyses of (**A1**) (**B1**) Na, (**A2**) (**B2**) P, (**A3**) (**B3**) V, (**A4**) (**B4**) O, and (**A5**) F on the interfacial anode disassembled from the NVP//NVP cell using (**A**) commercial liquid electrolyte and (**B**) all-solid-state ferroelectric-engineered composite electrolyte, respectively

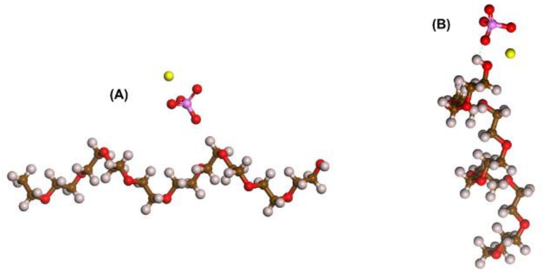

Fig. S11 Simulated interaction between $NaClO_4$ and (A) the repeated unit of PEO, and (B) the end group of PEO

To further analyze the interation between PEO and $NaClO_4$, quantum computing is calculated by Gaussian09 with B3LYP/6-311++G(d,p) basis set. Two interaction models are simulated. One is between $NaClO_4$ and the repeated unit of PEO and the other is between $NaClO_4$ and the end group of PEO. The interaction of $NaClO_4 \cdots$H-C (repeated unit) is relatively weaker, with an interaction strength of ~24.2 kJ/mol, but still much higher than the typical Van Der Waals interaction. On the other hand, the interaction between $NaClO_4$ and the end group (-OH) is higher, with a calculated strength of 73.7 kJ/mol, and therefore could be classified as a strong hydrogen bond.

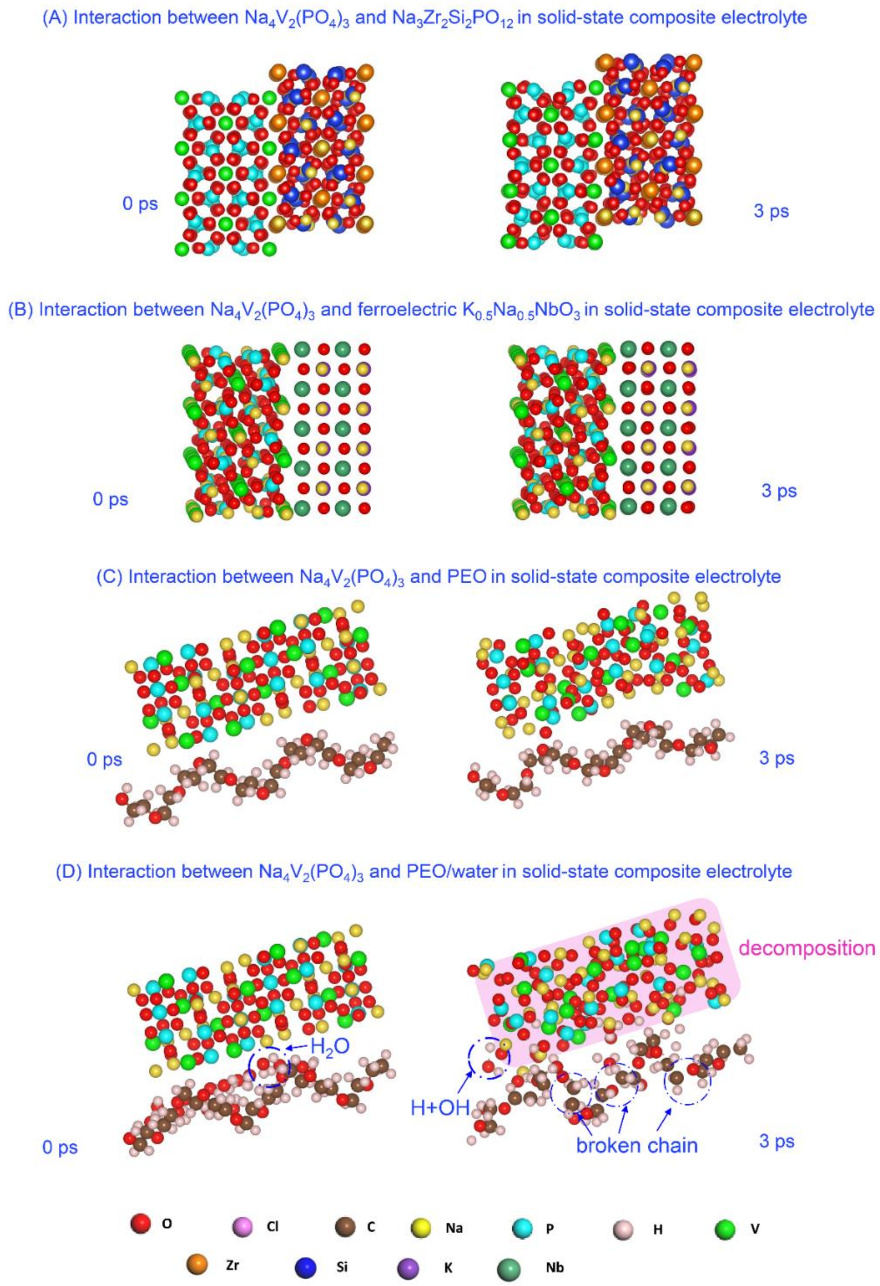

Fig. S12 Simulated interaction between the NVP anode and (**A**) $Na_3Zr_2Si_2PO_{12}$, (**B**) $K_{0.5}Na_{0.5}NbO_3$, (**C**) PEO, and (**D**) simulated interaction between the NVP anode and PEO with the appearance of some water molecules

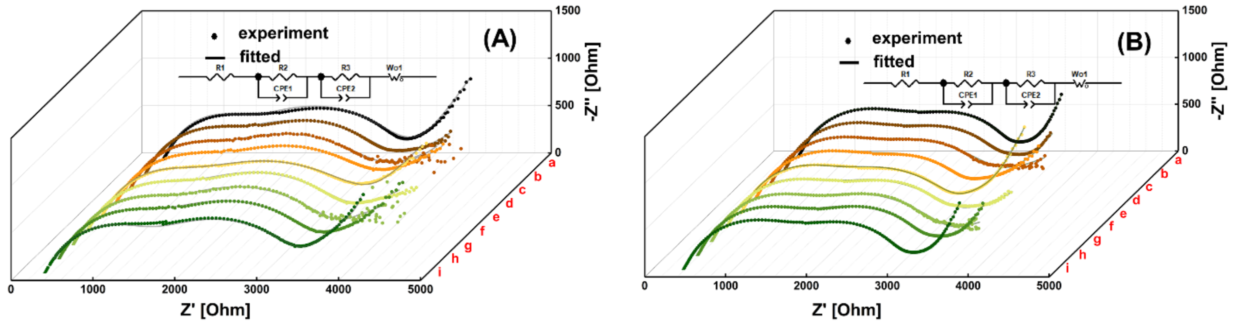

Fig. S13 Impedance plots of the all-solid-state NVP//NVP symmetric cells using (**A**) non-engineered composite electrolyte and (**B**) ferroelectric-engineered composite electrolyte, before/at/after the redox peak potentials

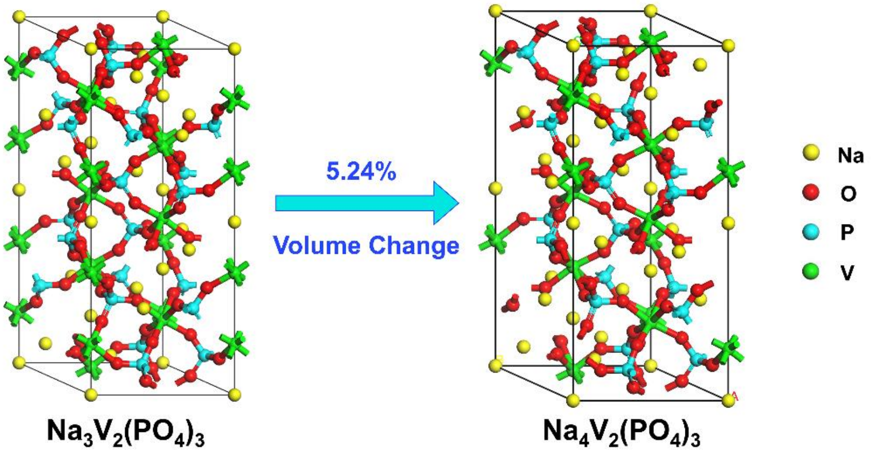

Fig. S14 Structure change of the NVP anode during charge/discharge based on DFT calculation
